# Diagnostics of Large Non-Conductive Anti-Corrosion Coatings on Steel Structures by Means of Electrochemical Impedance Spectroscopy

**DOI:** 10.3390/ma14143959

**Published:** 2021-07-15

**Authors:** Tomasz Jaśniok, Mariusz Jaśniok, Artur Skórkowski

**Affiliations:** 1 Faculty of Civil Engineering, Silesian University of Technology, 5 Akademicka, 44-100 Gliwice, Poland; tomasz.jasniok@polsl.pl; 2 Faculty of Electrical Engineering, Silesian University of Technology, 10 Akademicka, 44-100 Gliwice, Poland; artur.skorkowski@polsl.pl

**Keywords:** steel structures, anti-corrosion coatings, diagnostics, NDT, electrochemical impedance spectroscopy, EIS

## Abstract

This paper proposes a testing methodology for barrier properties of large non-conductive anti-corrosion coatings on steel structures. Electrochemical impedance spectroscopy (EIS) was adapted to in situ testing of steel structures by using a prototypical flexible measuring probe and a gel electrolyte that filled the probe, to take measurements on any surface regardless of its position. The first stage of the testing methodology was to perform time-consuming impedance measurements and quick electromagnetic measurements of coating thickness at selected test points. The results were used to determine correlation relationships between the logarithm of the impedance modulus for the coating at a measuring frequency of 0.1 Hz measured with the EIS method and the average thickness of the coating measured with an electromagnetic thickness gauge. Quick electromagnetic measurements were performed in the second stage to specify thickness of the other surface of the steel structure coating. The barrier properties of this coating were identified on the basis of the determined correlation.

## 1. Introduction

Steel structures are exposed to gradual corrosive degradation due to the impact of aggressive environments [1]. The most common anti-corrosive method for steel structures is to apply a (non-conductive) paint coating on its surface [2,3,4]. Thickness of this coating, which is an element used to verify the correctness of its application, can be measured during its application—WFT (wet film thickness), using combs or discs. The coating thickness can be also verified when the coating is dry. Then DFT (dry film thickness) gauges are used. These gauges usually measure thickness using the principle of magnetic induction or the eddy-current method [5]. Unfortunately, durability of this coating is also time-restrained, and its regular renewing is necessary [6,7]. Identifying the moment at which the paint coating lacks its proper barrier properties and its protective properties against corrosion are reduced is crucial for economic and durability reasons [8,9].

Conditions of paint coatings on typical steel structures are evaluated visually on the basis of ISO standards [10,11,12,13,14,15] or equivalent ASTM standards [16,17], which define, among other things, the method of evaluating the degree of rusting against the reference images. In case of critical structures, e.g., pipelines or buried tanks, conditions of anti-corrosion coatings are specified according to the standard [18], which states that electrical breakdown resistance is to be verified before installing such structures in the ground.

The above-mentioned economic aspect for maintenance of anti-corrosion coatings should not be based on the above qualitative data, but should identify the degradation of a coating, which requires quantitative methods. Electrochemical impedance spectroscopy (EIS) is such a quantitative method that can be used to determine conditions of the non-conductive anti-corrosion coating on metal surfaces [19,20].

**Figure 1:**
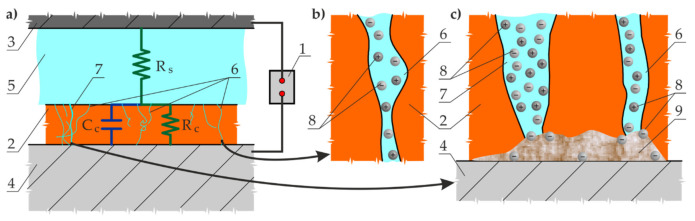
Scheme of impedance measurements of paint coatings on basic metal: (**a**) cross-section of paint-coated fragment of metal; (**b**) moisture and ions in the coating pore, the presence of which cause the current flow; (**c**) an increase in the number of pores and defects in the coating during localised corrosion, resulting in an increased conductivity. 1—analyser; 2—paint coating; 3—electrode; 4—coated metal; 5—electrolyte solution; 6, 7—pores and defects in the coating; 8—ions; 9—corrosion products; *R_s_—*impedance of electrolyte; *R_c_*—impedance of coating; *C*_c_—coating capacitance.

This method involves an analyser (1) to measure impedance *R_c_* for the coating *(*2) when alternating current flows between the electrode (3) and painted metal (4) within a wide range of frequencies (Figure 1a). An electrolyte layer (5) with resistance *R_s_* between the electrode (3) and the coating (2) provides the conductivity of the system. Current flow is possible in the coating (2), pores (6), and damaged areas (7) due to the motion of ions (8) in moisture. Ions (8) are usually from wet external environments and water-soluble electrolytes, which are components of the coating, and from impurities left after improper cleaning of metal prior to painting (Figure 1b,c)—cf. [21]. Ions (8) can serve as strong depolarizers (e.g., chloride or sulphate ions). Then, ions close to the metal surface (5) initiate corrosion in the presence of moisture and oxygen; the corrosion develops below the surface by creating a system of galvanic and concentration cells [1]. Due to oxidation, erosion, and coating aging, as well as accumulation of corrosion products (9) under the coated surface (Figure 1c), the coating shows more defects with time, which reduce its integrity and increase conductivity, which can be measured using the EIS method.

Electrochemical impedance spectroscopy is a method used to measure the coating impedance *R_c_* within a wide range of frequencies, and the capacitance response expressed as capacitance *C*_c_ or the constant phase element (CPE). By defining the so-called equivalent electrical circuits for the system tested (Figure 1a), numerical parameters can be determined for the model by adjusting the shape of the model spectrum to the determined one. It should be emphasized that moisture absorption by the coating or its gradual separation from the metallic substrate can be evaluated by analysing the EIS measurements [22].

The impedance values of visually satisfactory coating can be attributed to low, average, high, or very high barrier properties (cf. Table 1) following the recommendations specified in [23]. It must be pointed out that the standard [24] for testing paint coatings with the EIS method describes the recommended measurement methodology and the specification of the measuring system, but does not specify criteria for evaluating such tests.

There are many published papers on the EIS tests performed on non-conductive coatings applied on metals [25,26,27,28,29]. However, the method of impedance spectroscopy is rarely used on critical steel structures. Similar EIS applications, which are rare, refer to concrete structures, particularly to evaluate the corrosion rate of steel reinforcement [30,31,32,33,34]. However, it is important to highlight that many years ago impedance measurements were successfully taken on coatings of large steel structures, predominantly bridges and viaducts [35]. The results demonstrated in [35] indicated a significant diversity in the quality of coatings on various types of bridge structures, but reliable results and performance recommendations could not be presented, as there were too few impedance measurements. A time-consuming procedure is a drawback of this type of measurement, hence the limited time for the structure inspection results for few test points (as in [35]), and the consequently poor representativeness of the test results. The EIS method is time-consuming not only because a measurement itself usually takes longer than 10 min, but this procedure also requires that a cell be placed on the structure and then filled with an electrolyte solution of low resistance. The connection between the cell and the structure has to be tight to keep the solution in this cell, which is particularly difficult on uneven surfaces or in connections between structural components.

The objective of this article is to propose a methodology for testing barrier properties of large non-conductive anti-corrosion coatings, particularly on steel structures, using EIS. A flexible cell was proposed to take measurements, even at unequal connections between the steel elements. Tightness between the cell and the test surface was guaranteed by using conductive gel electrolyte. Within the framework of the developed methodology, the measurements of the thickness of the anti-corrosion coating with the quick method of magnetic induction should precede the selection of test points for the EIS testing, as provided in the standard [36]. Taking into account that, according to the standard [10], an increase in thickness of the tight coating to a specified value results in its higher barrier properties, a satisfactory correlation between these two methods was assumed to produce contour line maps of barrier properties of the anti-corrosion coating on the whole structure. In the end, having a very precise image of wear of the anti-corrosion coating, particularly on critical structures or elements with a poor access, the most economic or the least time-consuming process of the coating maintenance can be planned using various optimizing methods, as specified in, inter alia, the studies in [37,38,39].

## 2. Proposed Methods to Diagnose Large Anti-Corrosion Coatings

As already has been mentioned, the proposed methodology for testing barrier properties of large non-conductive anti-corrosion coatings on steel elements was based on the correlation between the electrochemical spectroscopy impedance (EIS) and magnetic induction. At first, the structure was divided into groups of elements having similar shape and the impact of the external environment; e.g., decks, webs of girders, bottom flanges of girder cantilevers, bracings, etc.

**Figure 2:**
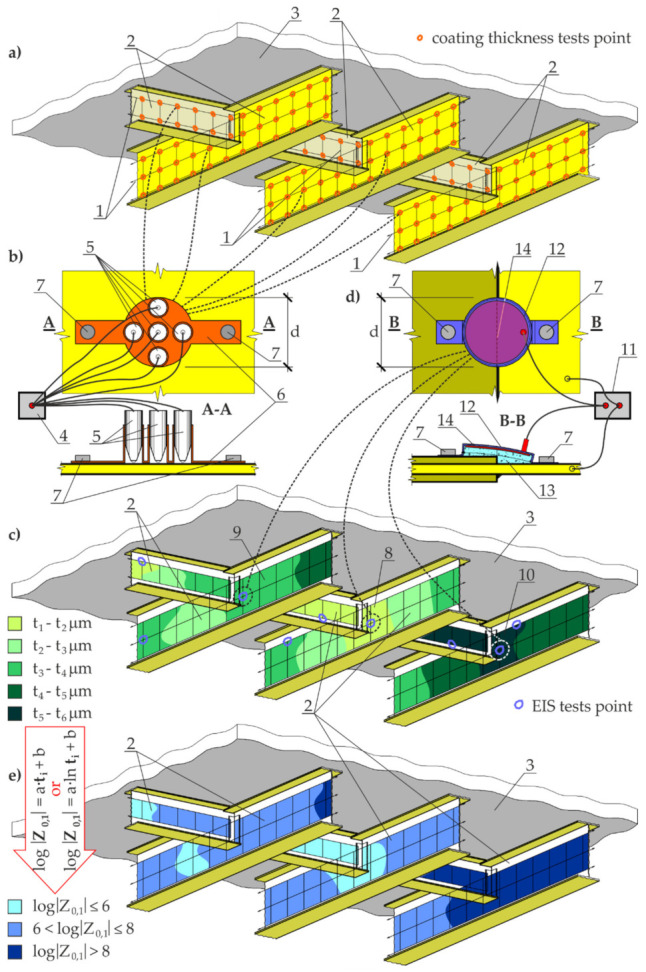
Procedure for identifying barrier properties of large anti-corrosion coatings: (**a**) the structure with the measuring grid and test points for measuring the coating thickness with the electromagnetic method; (**b**) the template to perform electromagnetic tests on thickness of the paint coating; (**c**) the contour line map illustrating the distribution of the coating thickness and the selected test points for the EIS; (**d**) the flexible head for the EIS testing; and (**e**) the contour line map illustrating the distribution of barrier properties of the paint coating defined on the basis of the coating thickness. 1—measuring mesh; 2—beam webs of concrete and steel floor selected for testing; 3—concrete floor slab; 4—coating gauge for the electromagnetic method; 5—measuring head; 6—template for testing the coating thickness using the electromagnetic method; 7—neodymium magnets; 8, 9, 10—areas with the lowest, moderate, and highest coating thickness, respectively; 11—potentiostat; 12—flexible cell; 13—conductive gel; 14—flexible auxiliary electrode.

Mesh arrangement and spacing (1) was individually defined for each group as shown in Figure 2a, using beam webs (2) of the standard steel and concrete industrial floor (3) as an example. Five measurements of the coating thickness were taken in each mesh node by the electromagnetic method using the gauge (4) with measuring heads (5) (Figure 2b). Spacing of the test points in the mesh node was restrained by a circular area with *d* diameter (Figure 2b), which could be best achieved using the template (6) fixed to the structure with the magnets (7). The magnets also provided the stability during the electromagnetic measurements, and the measurements could be easily taken simultaneously at the next node. The averaged values for five measurements of the coating thickness in the mesh node served as the basis for presenting the distribution of the coating thickness on the test elements in the form of the contour line map shown in Figure 2c.

Then, the tests were performed in the selected nodes using electrochemical impedance spectroscopy (EIS). This method required at least three test points within the areas with the lowest (8), moderate (9), and highest (10) thickness of the coating. Taking into account various thickness ranges, particularly the outliers, was very crucial for the EIS, as barrier properties of the coating usually improve as its thickness increases. On the other hand, the standard [10] specifies that exceeding a certain level of thickness can deteriorate mechanical properties of the coating and increase the retention time of the solution, which reduces good barrier properties of the coating. The EIS measurements were taken with the potentiostat (11) (Figure 2d). The standard [24] specifies the recommended technical parameters for this instrument. The proposed procedure did not include an option presented in [24], in which a cell with 3% NaCl solution was placed on the coating. The reason for such a proposal is a serious problem of ensuring tightness at the connection at an irregular increase in the surface thickness (also in the presence of screws, welds, rivets, and pads). For that reason, the cell (12) was filled at a small surplus with gel (13) based on 3% NaCl (as shown in Figure 2d). A cylindrical cell (12) was made of elastic plastic and contained the circular auxiliary electrode (14) made of stainless-steel sheeting. As in case of the template (6)*,* two magnets (7) stabilized the cell (12) and adjusted its shape to possible surface irregularities.

The next step was to determine the correlation between the impedance moduli $\left|Z_{0,1}\right|$ at a frequency of 0.1 Hz and the mean coating thickness *t* using arithmetic or a logarithmic scale. The value of 0.1 Hz was assumed as the frequency, at which aging properties of conductive and slightly conductive materials were revealed [40,41]. A higher coefficient of correlation determined the selection of a scale type for the variable *t*. If the correlation was not at least high for any of the analysed cases (the correlation could be assumed as high when *r* ≥ 0.5 [42,43,44,45]), the number of EIS measurements was increased until the required level of correlation was reached. However, the method was not representative if the coefficient *r* failed to increase despite an increasing number of the EIS measurements. After reaching the empirically determined value *r*, the relationship between the impedance modulus in logarithmic scale $\log\left|Z_{0,1}\right|$ and the coating thickness *t* in the previously specified scale was calculated as the value $\log\left|Z_{0,1}\right|$ at the individual nodes of the mesh. Thus, the criteria of evaluating the barrier properties of the coating (Table 1) developed on the basis of general recommendations given in [23] were used to draw the contour line map presenting the barrier properties of the examined anti-corrosion coating (Figure 2e).

**Table 1:** Criteria for evaluating the barrier properties of paint coatings on steel elements based on the logarithm of the impedance modulus *Z*_0,1_ at a measurement frequency of 0.1 Hz.

$log\left|Z_{0,1}\right|,\;\Omega {\mathrm{cm}}^{2}$	Barrier Properties
≤6	Low
6–8	Mean
8–10	High
>10	Very high

## 3. Materials

### 3.1. Test Materials

The tests were performed on three types of commercially available paint coatings applied on metal sheets made of S 235 steel of two thicknesses. The element composition of low-carbon steel of grade S 235 included C ≤ 0.23%, Mn ≤ 1.3%, Si ≤ 0.4%, P ≤ 0.07%, and S ≤ 0.065%. Two small metal sheets measuring 250 mm × 80 mm had a thickness of 2 mm, while one large metal sheet with dimensions of 2000 mm × 1000 mm was 5 mm in thickness. The substrate of each sheet for painting was prepared by blast cleaning according to the standard [46] to a cleanliness level of Sa 2½, and then acetone was used to clean and degrease the sheets.

**Figure 3:**
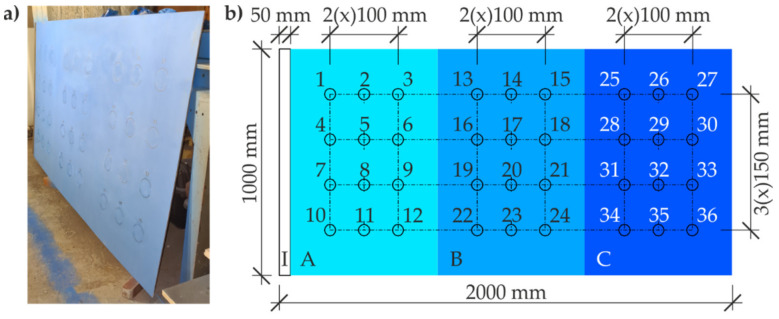
The steel sheet for main tests: (**a**) the sheet before testing; (**b**) the model divided into areas of different thickness of the paint coating and the numbered test points.

The commercially available claret-coloured coating with a thickness of 5–8 μm was pneumatically sprayed on the first of the small metal sheets. The other small metal sheet was protected with a commercially available grey-coloured coating with a thickness of 25–48 μm, which was applied with a paint roller. The large metal sheet was coated with a third type of the commercially available blue-coloured paint coating (Figure 3a). To differentiate the coating thickness, three layers of the paint were applied to this metal sheet by pneumatic spraying. At first, almost the entire sheet was covered with one layer of the paint, except for a strip of 50 mm in width at a shorter edge without the coating. This area was used to connect the potentiostat—area *I* in Figure 3b. The second layer was applied after 24 h (as specified by the manufacturer), but only to ca. 2/3 of the sheet surface. After other 24 h, the third, final layer was applied only to ca. 1/3 of the sheet surface. On this metal sheet, there were four areas in total: area *I* without the paint coating, area A with one layer of 50–70 μm in thickness, area B with two layers of 70–130 μm in thickness, and area C with three layers of 130–150 μm in thickness. A grid was put on each A–C area (with a mesh size of 100 mm × 150 mm). Twelve numbered test points were selected in each grid node, each ca. 60 mm in diameter.

All coatings were applied at an air temperature of 22 ± 2 °C and a relative humidity of 55 ± 5%. It should be emphasized that all three paint coatings (claret, grey, and blue) had a proprietary chemical composition. However, these tests did not focus on evaluating the quality of some coatings, but on testing the proposed methodology with reference to its effectiveness on each non-conductive paint coating on a steel surface.

### 3.2. Materials Used for Tests

The tests employed electrochemical cells that were filled with the electrolyte solution and had auxiliary electrodes. These cells were produced from ABS and Zortrax Z-Flex plastic of high flexibility and 31 ShD Shore hardness. The auxiliary electrodes were a film of 0.05 mm in thickness, cut from X5CrNi18-10 stainless steel to the disk shape.

Two types of electrolytes, aqueous and gel, were used in the tests. The aqueous electrolyte was 3% NaCl. The gel electrolyte was dense gel produced by dissolving guar gum (E412) in 3% NaCl aqueous solution. Proportion by weight of each gel component; that is, E412 and 3% NaCl, was 3:50. The expected consistency of the thick gel was obtained after 30–45 s of stirring.

## 4. Methods

In the first stage of the tests, the potential use of conductive gel as the electrolyte solution for the EIS testing was verified. The second stage included the tests on the coating thickness performed by the electromagnetic method in 36 test points in the nodes of the grid plotted on the large metal sheet with blue-coloured paint coating of three different thicknesses. Then, the EIS tests were conducted at the same test points using the conductive gel and the flexible electrochemical cell.

### 4.1. Selection of an Electrolyte Solution

The standard [24] does not specify requirements for the solutions for the tests on non-conductive anti-corrosion coatings on conductive materials by the EIS. Various solutions can be used, provided that their electrical resistance is low when compared to impedance of the test system. Moreover, the standard [24] recommends that the electrolyte selection is related to conditions of the coating exposure. Therefore, the electrolyte was selected in accordance with the standard [47], which specifies that corrosion resistance of popular metals can be determined from the information on the environment. The chloride environment is particularly hazardous to steel civil-engineering structures. These conditions are mainly in seaside areas and in the vicinity of road infrastructure during the winter deicing of roads. In addition, electrolyte solutions with chloride ions have good conductivity. This is why NaCl at the most corrosive concentration of 3%, as specified in [1], was used in the EIS tests. In addition, the gel specified in Section 3.2 was used in the tests.

**Figure 4:**
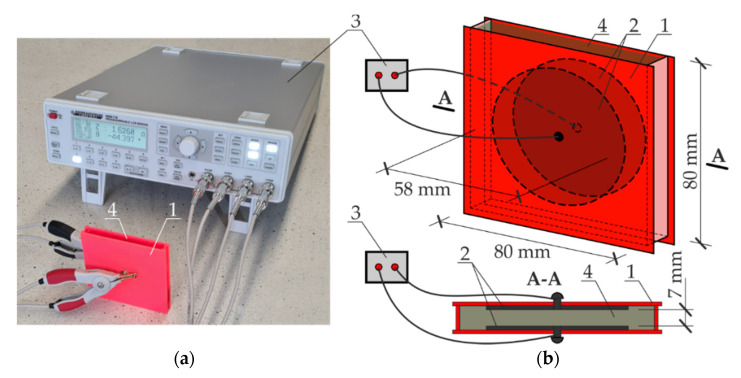
The test stand for measuring impedance of aqueous solution of liquid and gel electrolytes: (**a**) test stand, (**b**) design of the measuring cell. 1—measuring cell; 2—circular auxiliary electrode; 3—LCR measuring bridge; 4—liquid or gel electrolyte.

**Figure 5:**
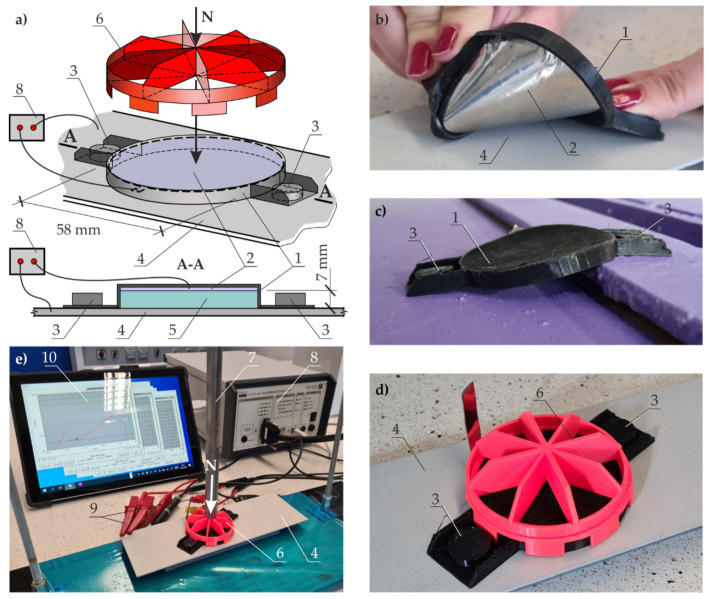
Measuring cell and the test stand in the first stage of EIS testing of anti-corrosion coatings: (**a**) schematic view of the measuring head on the sheet with paint coating; (**b**) head flexibility; (**c**) adjusting head shape to stepped connection between the test elements; (**d**) view of the head with a cap to conduct tests with liquid electrolyte; (**e**) view of the test stand and the head with a cap to conduct tests with liquid electrolyte. 1—flexible measuring cell; 2—flexible auxiliary electrode in the form of film made of stainless steel; 3—neodymium magnets; 4—sheet with paint coating; 5—liquid or gel electrolyte; 6—head cap for measurements with liquid electrolyte; 7—screw pressure; 8—high impedance analyser; 9—clamps for high impedance analyser; 10—hybrid laptop.

A small traditional conductivity cell used to measure conductivity of the gel does not guarantee that the whole cell is filled, and consequently, the conductivity measurement can be incorrect. Hence, a large measuring cell of our own construction, as illustrated in Figure 4, was used in the tests. The cell (1) made of ABS plastic had sufficient size to place round electrodes (2) that were 58 mm in diameter, 7 mm apart. A diameter of the electrodes and the steel grade were similar to dimensions of the electrode in the probe employed in the EIS testing (Figure 5). The distance between the electrodes was equal to the height of the probe.

The system impedance was measured with the LCR bridge (3) at a frequency of alternating current of 1000 Hz. The cell was filled with the solution (4). The measurements were at first taken in 3% aqueous solution of NaCl. After washing with distilled water and drying, the impedance of the gel with composition specified in Section 3.2 was measured.

### 4.2. Comparative Tests of Coating Impedance Using a Probe Filled with Liquid and Gel Electrolyte

A 3D-printed measuring cell, illustrated in Figure 5a, was used in the tests. The cell (1) bottom was made of Zortrax Z-Flex plastic, to which disk-shaped film (2) made from stainless steel was fixed. It had a diameter of 58 mm (the same diameter as that of the cell in Figure 4b) and a thickness of 0.05 mm. As the cell was made of flexible plastic and the electrode was made of thin film, the cell could be freely deformed, as shown in Figure 5b. This was particularly important for the tests performed on irregular surfaces, as well as the ones with gradually changing height (Figure 5c). The neodymium magnets (3) were used to stabilize the cell on the surface during measurements. The cell could be then stabilized regardless of the position of the test surface; that is, vertical or horizontal position.

In this stage of the tests, impedance was measured for two small steel sheets (4) (Figure 5) specified in Section 3.1. They were protected against corrosion with claret and grey coatings. Two types of electrolytes (5) were used as specified in Section 3.2. Tightness between the test surface and the cell walls was required for the liquid electrolyte (3% NaCl). For that purpose, a cover plate (6) (Figure 5a,d,e) made of ABS was used to stiffen the cell. The screw (7) pressure was applied to the plate (6), and the force *N* increased until the full tightness of the cell was achieved (Figure 5a,e). In the case of the gel electrolyte (E412 in 3% NaCl solution), the cell tightness was not necessary, as gel was inside throughout the whole period of measurements. The gel electrolyte, as the aqueous electrolyte, was used to provide the flow of polarizing current between the auxiliary electrode (2) and the working electrode (4) in the form of the sheets with paint coatings. The described two-electrode setup was connected to the high impedance analyser (8) by a ATLAS 0441 HIA with clamps (9) (Figure 5e). The analyser was controlled with a hybrid laptop (10). Impedance was measured within a frequency range of 0.1 Hz–100 kH, at the steady potential amplitude of 20 mV. These tests were performed to evaluate the possibility of replacing the traditional aqueous electrolyte with gel electrolyte in measurements of polarization of paint coatings applied to large elements of steel structures.

### 4.3. Measurements of Coating Thickness by Electromagnetic Method with Template

**Figure 6:**
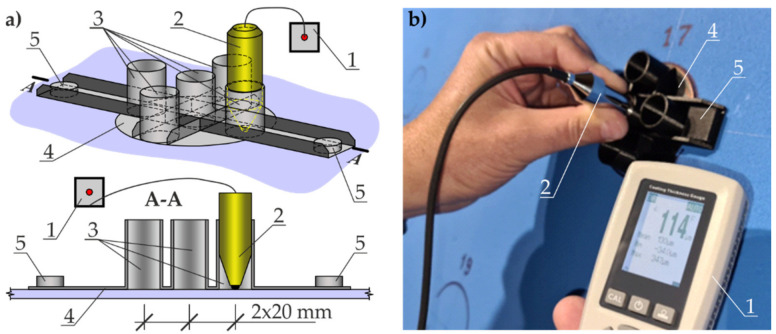
Test stand for measuring thickness of the paint coating by the electromagnetic method: (**a**) diagram of the measuring template; (**b**) view during the measurement taken on the sheet. 1—inductive thickness gauge; 2—measuring head of the thickness gauge; 3—head guides; 4—the measuring pattern; 5—neodymium magnets.

The second stage of the tests was conducted on the large metal sheet coated with a commercially available blue-coloured paint coating, as specified in Section 3.1 (Figure 3). Five measurements of the paint-coating thickness were taken at each test point in the grid nodes by the electromagnetic method with the inductive thickness gauge (1) (Testan DT-25 FN, Alfatech, Cracow, Poland). This instrument analysed measurement signals from the head (2) placed in five different guides (3) in the measuring pattern (4) illustrated in Figure 6. The measuring pattern (4) stabilised on the sheet surface with neodymium magnets (5) guaranteed a stable spacing of the test points equal to 20 mm. The thickness gauge (1) was used to average measurements for a given measured area. Average measured values were the base to illustrate distribution of the coating thickness on the test steel sheet.

While placing this type of head in the measuring pattern (4), attention was paid to the effect of the electromagnetic field of the neodymium magnets (5) that fixed the templates to the steel sheet (Figure 6). The position of two magnets in relation to the measuring head was corrected during the tests to eliminate this effect.

### 4.4. Measurements of Coating Impedance by the EIS with a Probe Filled with Gel Electrolyte

**Figure 7:**
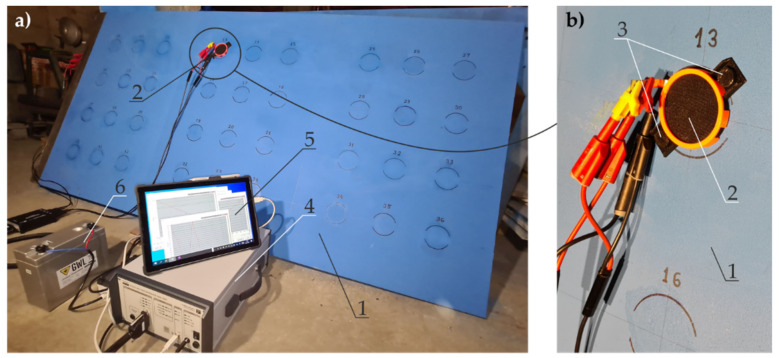
A stand for the EIS testing with a probe filled with gel electrolyte: (**a**) steel sheet with paint coating, the instrument, and the measuring head; (**b**) the measuring head. 1—test sheet of 2000 × 1000 mm surface area with blue coating; 2—measuring head; 3—neodymium magnets of the head; 4—high impedance analyser; 5—hybrid laptop; 6—battery.

The second main stage of the impedance tests was conducted on the large metal sheet with a commercially available blue-coloured paint coating applied to the same test points as specified in Section 4.3. Thickness of coatings was measured by the electromagnetic method. Impedance was measured in each of 36 test points with the flexible head, the inside part of which was covered with film (Figure 5b) and served as the auxiliary electrode. The measuring head was filled only with the gel electrolyte specified in Section 3.2. The sheet coating (1) was tested in the upright position (Figure 7a), which allowed us to verify the performance of the measuring head (2) fixed with neodymium magnets (3) and filled with thick electrolyte gel, which did not flow from the head (Figure 7b).

Impedance was measured as in the first stage of the EIS tests using the high-impedance analyser (4) (ATLAS 0441 HIA, Atlas-Sollich, Gdansk, Poland) controlled with the hybrid laptop (5). The device was charged with the battery (6) to avoid any measurement disturbances. The EIS test was conducted in a two-electrode arrangement, in which the working electrode was the sheet with a paint coating, and the auxiliary electrode was stainless-steel film in the measuring head. Impedance was measured at the range of frequencies of 0.1 Hz–100 Hz at a steady potential amplitude of 20 mV.

## 5. Results

### 5.1. Comparative Tests of Conductivity of Aqueous and Gel Electrolyte

The measured impedance of the 3% NaCl aqueous solution and gel, the composition of which was as specified in Section 3.2, indicated that the impedance values were very similar. The absolute impedance of gel layer having an area of 2642 mm^2^ and a thickness of 7 mm was 1.8 Ω, while the absolute impedance of the 3% layer of aqueous NaCl of the same size was 1.7 Ω (a ca. 6% difference).

### 5.2. Comparative Tests of Coating Impedance Using the Probe Filled with Aqueous and Gel Electrolyte

**Figure 8:**
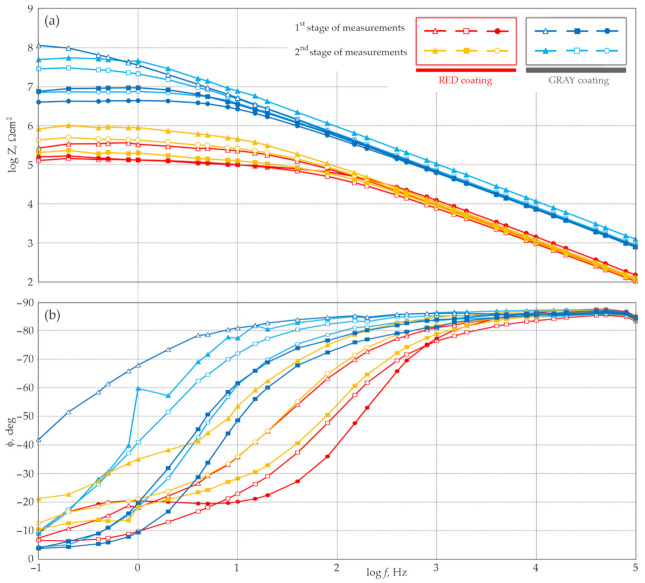
Measured impedance for two small sheets with a thinner red coating and a thicker grey coating: (**a**) the Bode plot Z(f); (**b**) the Bode plot φ(f)—described in the text.

Data from measured impedance are presented on the Bode plots illustrated in Figure 8. On the diagram of modulus of impedance *Z* and measurement frequency *f* (Figure 8a), groups of spectra characteristics similar in shape for both tests sheets can be noticed. In case of the sheet with a thinner red layer, the logarithm of impedance modulus was within the range of 5–6 Ωcm^2^, whereas for the sheet with a thicker grey layer, the modulus was within a higher range of 6.7–8.1 Ωcm^2^. The assessment of protective properties of the test coatings based on Table 1 showed that the red coating had low barrier properties, and the grey coating had moderate ones. Observations based on the analysis of the plot in Figure 8a were also confirmed on the Bode plot (Figure 8b)—by the relationships between the angle of phase shift *φ* and the measurement frequency *f*.

A clear drop in the phase-shift angle over reducing frequency of the measuring signal was noticed for both coated sheets. The drop for the thinner red coating was faster than in case of the sheet with grey coating. This meant that the barrier properties of the thinner (red) layer were worse than expected when compared to the thicker (grey) layer.

However, the presented qualitative evaluation of coatings based on measured impedance was not the principal aim of this experiment. The tests were performed to find whether the traditional aqueous electrolyte could be replaced with the gel electrolyte in measuring impedance of coatings. For better clarity, individual points of the impedance spectrum (circles, triangles, or squares) on the Bode plots (Figure 8) measured with the gel electrolyte are coloured. The spectrum points measured with the aqueous electrolyte have the same shapes, but are not filled with colour. Blue and red used for both groups of spectra indicate the first series of measurements taken the same day at 15 min intervals. Orange and light blue colours represent spectra taken in the second series of measurements performed after 24 h.

By analysing shapes of impedance spectra for these three groups of colours, no significant differences in the impedance modulus |*Z*| can be noticed in Figure 8a, regardless of the use of aqueous or gel electrolytes. The plot in Figure 8b also does not indicate any sign that one of the test electrolytes could affect the shape over the frequency of the phase shift angle *φ*.

### 5.3. Measurements of Coating Thickness by Magnetic Induction

**Figure 9:**
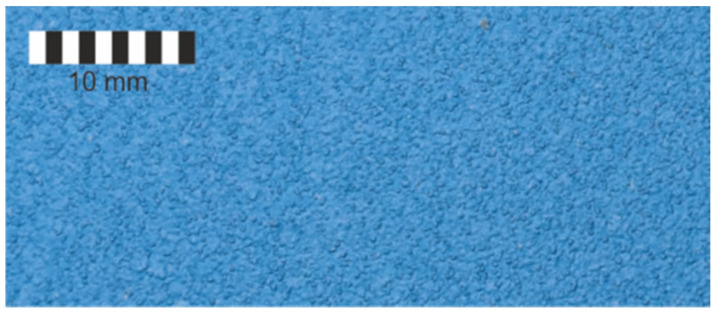
Image of the rough structure of the paint coating affecting the range of thickness measured by the electromagnetic method.

Thickness of paint coatings at all 36 test points (Figure 3b) was determined as the mean result computed by the software. This value was obtained from five readings of thickness at the points selected with the template placed on the sheet surface (Figure 6). Thickness of the paint coating in the areas A, B, and C was not the same based on the assumed preparation of the surface of the steel structure, on which some minor impurities were acceptable, and also due to roughness of the coating surface illustrated in Figure 9. Measured mean thickness of the coating in a given area instead of a single reading was to eliminate an accidental characteristic error for the test points.

Five measurements in each test area determined the mean thickness of the paint coating and also the standard deviation. Based on the measurements, the standard deviation could be used as a parameter defining the quality (uniformity) of the test paint coating.

**Figure 10:**
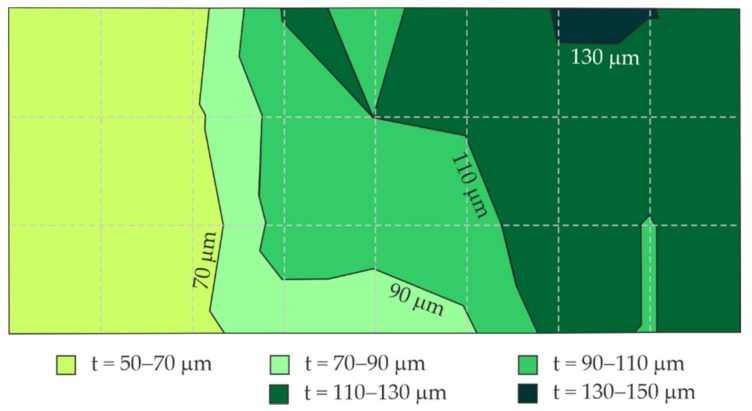
Map of thickness distribution of blue coating on the large steel sheet based on measurements with the gauge.

Figure 10 presents a distribution of the blue coating thickness on the large steel sheet (Figure 3) obtained from the measured mean thickness of the coating at particular test points, including their location (nine columns, four rows).

### 5.4. Measurements of Coating Impedance by the EIS with a Probe Filled with gel Electrolyte

**Figure 11:**
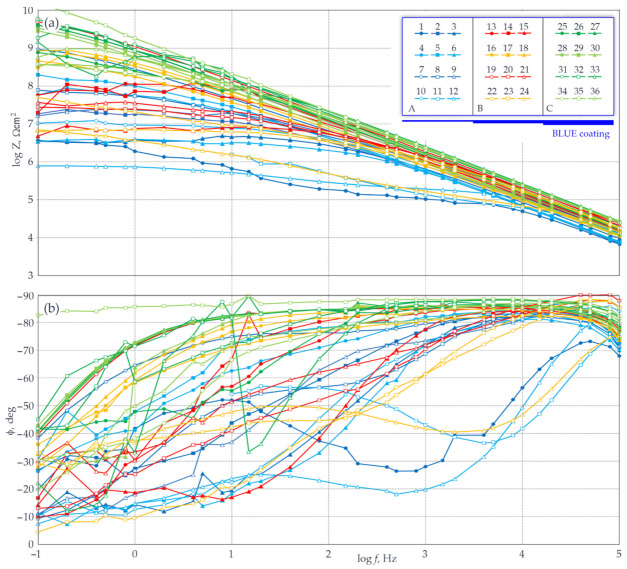
Measured impedance for the large steel sheet with the blue paint coating and the areas A, B, and C of different thickness of the coating: (**a**) the Bode plot Z(f); (**b**) the Bode plot φ(f)—described in the text.

The measured impedance for the large steel sheet (Figure 3) painted with one, two, or three blue coatings, is shown in Figure 11. The EIS tests were performed with the probe filled with the gel electrolyte specified in Section 4.2. For better clarity of the presented results, the plots of impedance spectra contain three main groups of colours (blue, orange-red, and green) for spectra obtained for the sheet with one (A), two (B), or three (C) layers of the paint coating. Different shapes and colour patterns shown in the plots were attributed to rows and columns of individual test points.

The analysis of the shapes of impedance spectra shown on the Bode plot (Figure 11a) indicated that as expected, the highest impedance moduli for the characteristic measurement frequency of 0.1 Hz were obtained for three layers of the paint coating. The logarithm of the impedance modulus Z_0.1_ for green and light-green spectra was within the range of 8.5–10.3 Ωcm^2^. It should be noted that values of the modulus Z_0.1_ for the frequency 0.1 Hz that exceeded 8 Ωcm^2^ clearly implied good barrier properties of the test coating (Table 1). Additionally, based on the analysed variable path of the curve as a function of the phase-shift angle, its slow drop could be observed within the low-frequency range. For the majority of cases at 0.1 Hz, the achieved values were φ_0.1_ > 40° (Figure 11b). An exceptionally high value of φ_0.1_ = 83° was found only in one case. For four spectra, the angle φ_0.1_ reached relatively low values within the range of 20–31°. The paint coatings generally having typical angles φ_0.1_ > 45° within a wide range of measurement frequencies were considered to ensure good anti-corrosion protection for steel.

The spectra illustrated on the Bode plots (Figure 11a) in red and orange colours describe the central area of the test sheet (B) with two layers of the paint coating. The analysed distribution of the test points in the upper part of the Bode plot (Figure 11a) evidently demonstrated that the logarithms of the impedance moduli for 0.1 Hz were within a wider, and at the same time the lowest, range of values (Z_0.1_ = 6.7–9.7 Ωcm^2^) than the values read for three layers of the paint coating. Drops and disturbed path of a change in the phase-shift angle indicated worse barrier properties of the two-layer coating at higher frequencies (>10 kHz). The angle values φ_0.1_ read at the frequency 0.1 Hz were rather uniformly distributed within a wide range of 5–40°. This range of values was clearly lower than the one obtained for three layers of the blue coating.

The final analysed group of spectra in blue and light-blue colour describe the third area (C) of the test sheet with only one layer of the paint coating. The Bode plot shows low barrier properties of the weakest by default, because it was the thinnest coating layer (Figure 11a). In this case, the spectra describing the impedance moduli were not close to each other. Consequently, there was a significant dispersion of the logarithms of the impedance modulus, which were equal to Z_0.1_ = 5.8–8.9 Ωcm^2^ at the frequency 0.1 Hz. Worse barrier properties of one layer of the coating were also shown on the Bode plot φ(f) (Figure 11b), on which sudden drops in the phase angle and in measurement frequencies were evident. Phase-angle drops for the majority of blue and light-blue spectra began at 1 kHz, and for three spectra even below100 kHz. Dispersion of these values was similar to the two-layer coating and was within a slightly narrower range of 7–39°.

**Figure 12:**
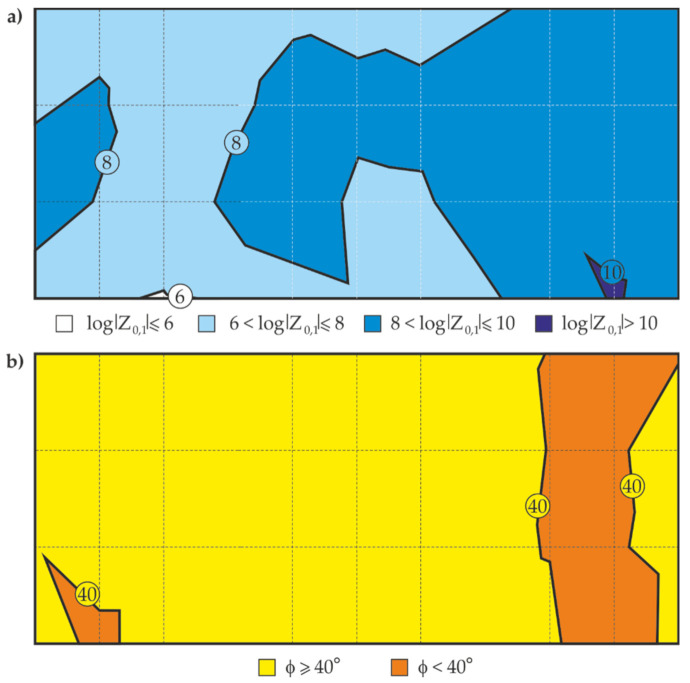
Maps of distribution for the blue coating on the large steel sheet: (**a**) the logarithm of impedance modulus Z_0.1_, (**b**) the phase-shift angles φ_0.1_, for the measurement frequency of 0.1 Hz.

Similarly to the thickness measurements by the electromagnetic method, Figure 12a shows the maps of distribution of the logarithm of impedance modulus Z_0.1_, and Figure 12b presents the maps of distribution of phase-shift angle φ_0.1_, determined at the test points with reference to their location (nine columns and four rows).

## 6. Discussion

Table 2 shows presents mean values of thickness *t* of the blue paint coating determined at the test points 1–36 on the large steel sheet. They are mean values based on five measurements using the template shown in Figure 6a. Standard deviations from measured values (numbers with the ± sign in Table 2) were also determined for each test point. Table 3 and Table 4 summarise the values of logarithm of impedance modulus Z_0.1_ and phase-shift angles φ_0.1_ determined from the measurements taken at the above points on the sheet by the EIS method.

The measured values shown in Table 2, Table 3 and Table 4 refer to the same test points 1–36 arranged on the test steel sheet as illustrated in Figure 3b. Estimates *r_xy_* of the correlation coefficient for two variables *x*, *y* [48] were calculated using Equation (1) with the *z* bias estimate of covariance and standard deviations for the values shown in Table 2 and Table 3 (*x* = *t*, *y* = log|*Z*_0.1_|) and Table 2 and Table 4 (*x* = *t*, *y* = φ_0.1_). According to the proposed methodology described in Section 2, calculations in the second step were repeated for the same data from Table 2, Table 3 and Table 4, but for the variable *x* = ln *t*:

(1) $$r_{xy}=\frac{m_{o,xy}}{s_{x}\;s_{y}}$$

Bias estimates *s_x_* and *s_y_* (empirical variations) of standard deviations *σ_x_* and *σ_y_* were determined using Equation (2):

(2) $$s^{2}=\frac{1}{n}\sum_{i=1}^{n}{\left(x_{i}-\bar{x}\right)}^{2}=\frac{1}{n}\sum_{i=1}^{n}x_{i}^{2}-{\left(\frac{1}{n}\sum_{i=1}^{n}x_{i}\right)}^{2},$$

whereas the bias estimate of the covariance was calculated using Expression (3):

(3) $$m_{o,xy}=\frac{1}{n}\sum_{i=1}^{n}(x_{i}-\bar{x})(y_{i}-\bar{y})=\left(\frac{1}{n}\sum_{i=1}^{n}x_{i}y_{i}\right)-\bar{x}\cdot \bar{y}.$$

**Figure 13:**
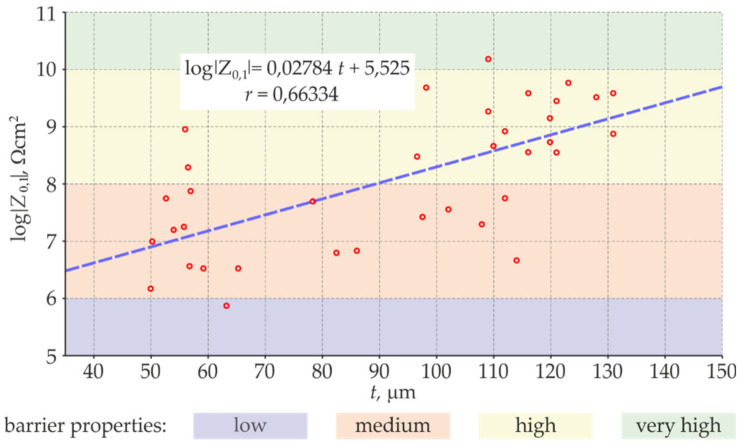
Correlation relationships between the thickness *t* of the blue coating and the logarithm of impedance modulus *Z*_0.1_ determined at 36 test points.

The estimate *r_xy_* of the correlation coefficient was calculated on the basis of series of measured variables *x*_i_ and *y*_i_, where *i* = 1, …, 36 (data for all test points), and *n* was the size of the series (*n* = 36). The values *x* and *y* are mean values per series. The *Statistica* software and [49] were used to analyse the correlation of the measured values to verify their consistency, taking into account the average thickness of the coating *t* in arithmetic scale (*x* = *t*) and logarithmic scale (*x* = ln *t*). Better correlation was found for the variable *x* = *t.* The further analysis as specified in Section 2 was conducted only for this variable. Figure 13 presents the dispersion of correlation relationships between the thickness *t* of the blue coating determined by the electromagnetic method and the logarithm of impedance modulus *Z*_0.1_ determined by the EIS method.

This diagram shows a true linear relationship between the analysed variables. The estimate of the correlation coefficient r = 0.663 obtained in this case indicates, according to [42,43,44,45], high correlation and a significant relationship between analysed values determined by different methods.

**Figure 14:**
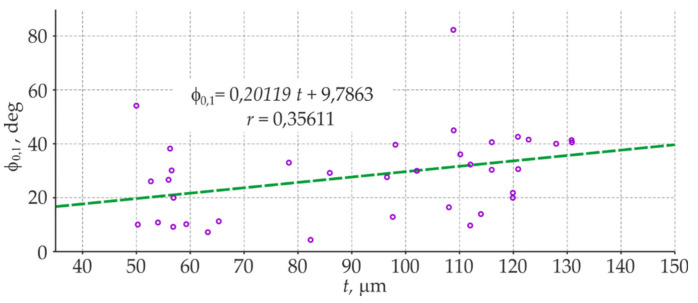
Correlation relationships between the thickness *t* of the blue coating and angles of the phase shift *φ*_0.1_ at 36 test points.

Figure 14 presents the correlations between the thickness *t* of the blue coating determined by the electromagnetic method and the phase-shift angle *φ*_0.1_ determined by the EIS method. The obtained estimate of the correlation coefficient *r* = 0.356 indicated the low correlation and only clear relationship between the thickness of the coatings and the angles of phase shift. The insignificant level of the correlation excluded the phase-shift angle as the second parameter, which could be potentially taken into account in the methodology for testing barrier properties of large non-conductive anti-corrosion coatings applied to steel structures.

**Figure 15:**
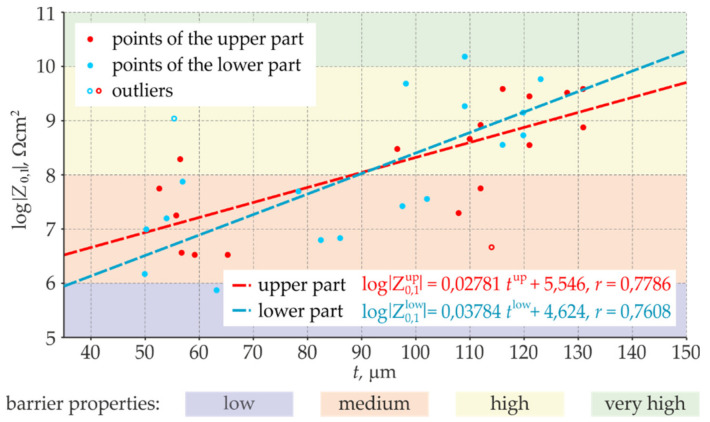
Correlation relationships between the thickness *t* of the blue coating and the logarithm of impedance modulus *Z*_0.1_ were determined separately for the upper and lower parts of the sheet (in red and blue, respectively, for points, lines and empirical equations).

**Figure 16:**
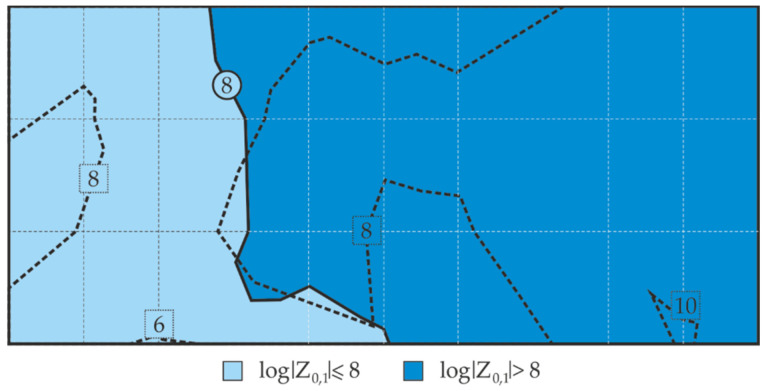
Map of distribution of the logarithm of impedance modulus *Z*_0,1_ calculated from the correlation relationships given in Figure 15, based on measured thickness *t* of blue coating on the large steel sheet.

According to the methodology described in Section 2, the barrier properties should be calculated based on the empirical relationship shown in Figure 13. However, it can be done after determining high correlation between the thickness *t* of the blue coating determined by the electromagnetic method and the logarithm of impedance modulus *Z*_0.1_ determined by the EIS method. For better verification of the developed methodology, it was decided to calculate the barrier properties of the points in the lower part of the sheet (rows 3 and 4 in Table 2 and Table 3) on the basis of the correlation of the points in the upper part of the sheet (rows 1 and 2 in Table 2 and Table 3). The outlier points were eliminated prior to the calculation, and defined empirical relationships are illustrated in Figure 15—the equation is shown in red colour. The empirical relationship was similarly determined from the points in the lower part of the sheet using data from the rows 3 and 4 in Table 2 and Table 3. This relationship, marked in blue colour in Figure 16, was used to determine the barrier properties of the coating in the upper part of the sheet (rows 1 and 2).

The calculated results are presented in Table 5. Relative percentage change against the value directly measured with the EIS is given in brackets at each calculated value of the logarithm of the impedance modulus. With reference to Table 3 presenting the measured logarithms of the impedance moduli, the number expressed in (%) at the calculated values of log|*Z*_0,1_| in Table 5 means lower value (−) or higher value (+). A difference between the measured and calculated logarithm of the impedance moduli did not exceed 10% for as many as 22 test points. For seven tests points, the calculated log|*Z*_0,1_| changed a class of barrier properties defined in Table 1. With reference to the measured values, the barrier properties determined from the correlation relationships were lower by one class in five cases (yellow cells in Table 5), and in two cases they were higher (blue cells in Table 5).

Differences between calculated and measured barrier properties of the examined coating are shown in the contour line map of the distribution of the impedance modulus logarithm in Figure 16. The analysis of this map in Figure 16 indicated that only two areas determined from the calculated logarithms of impedance moduli Z_0.1_ had moderate and high barrier properties in accordance with the criteria specified in Table 1. Boundary lines of the areas determined by the indirect measurements with the EIS were marked on the contour line map with a black broken line for comparative purposes (cf. Figure 12a). There is an evident convergence of the localization of the areas with the same barrier properties and the path of the boundary lines between these areas. Hence, it can be concluded that the distribution of the barrier properties determined from the calculated values generally did not change with reference to the measured values. This could indicate that the proposed methodology is applicable to technical evaluation of barrier properties of paint coatings in non-destructive in situ tests conducted on large steel structures.

## 7. Conclusions

On the basis of the conducted tests and the numerical analysis, the following conclusions could be drawn:

- The method of electrochemical impedance spectroscopy can be used not only for testing non-conductive coatings on metals under laboratory conditions, but also, after the required adaptation, for testing anti-corrosion coatings on large elements of steel structures.
- Adaptation of the EIS to the in situ tests was mainly based on using the flexible housing of the measuring probe with the integrated flexible auxiliary electrode, the shape of which adjusted to the test surface, and using electrolyte gel instead of the traditional aqueous electrolyte.
- The coating thickness measured at the same test points with the EIS and electromagnetic gauge (average values of a few measurements) demonstrated a significant relationship and a high correlation between the logarithm of the impedance modulus and the average thickness of the coating. The specified relationship between the phase-shift angle and the mean coating thickness had a low correlation, and thus could not be used as an auxiliary parameter in this methodology.
- Following the proposed measurement methodology, a high correlation between the logarithm of the impedance modulus and the mean thickness of the anti-corrosion coating was obtained for parts of the test steel structure and was used to determine the empirical relationships between these parameters. Then, the distribution of the barrier properties of the non-conductive anti-corrosion coating on the whole surface of the test steel structure could be determined on the basis of nothing but quick measurements of the coating thickness.
- The proposed test procedure is currently at the stage of preliminary tests and requires further measurements and analyses. In particular, the tests on the effect of different types of non-conductive coatings and their thickness are required, and on the recommended number of the test points and the number of measurements of the coating thickness at each test point. On the other hand, the presented results and adaptive details of the EIS method for testing large steel structures indicated that this methodology can be recognised as a quantitative method of testing anti-corrosion coatings that is relatively quick compared to currently applied qualitative methods.

## Figures and Tables

**Table 2:** Comparison of mean thickness values *t* (μm) of the coating at 36 test points on the large steel sheet with the blue coating.

Coating	1 Layer			2 Layers			3 Layers		
Row/column	1	2	3	4	5	6	7	8	9
1	57 ± 9	56 ± 11	59 ± 3	112 ± 4	108 ± 9	114 ± 5	131 ± 4	131 ± 7	116 ± 4
2	53 ± 8	57 ± 4	65 ± 15	97 ± 10	110 ± 10	112 ± 9	128 ± 8	121 ± 8	121 ± 9
3	56 ± 3	57 ± 4	54 ± 4	98 ± 7	98 ± 12	102 ± 6	123 ± 8	109 ± 2	120 ± 1
4	50 ± 7	50 ± 2	63 ± 9	82 ± 4	78 ± 4	86 ± 6	116 ± 12	109 ± 6	120 ± 10

**Table 3:** Comparison of logarithms of the impedance moduli |*Z*_0.1_| (Ωcm^2^) at 36 test points on the large steel sheet with the blue coating at a measurement frequency of 0.1 Hz.

Coating	1 Layer			2 Layers			3 Layers		
Row/column	1	2	3	4	5	6	7	8	9
1	6.56	7.27	6.54	7.76	7.30	6.68	8.88	9.60	9.60
2	7.76	8.29	6.53	8.48	8.66	8.93	9.52	9.45	8.55
3	8.96	7.89	7.21	9.69	7.44	7.57	9.76	9.27	9.16
4	7.02	6.19	5.89	6.80	7.70	6.85	8.58	10.19	8.76

**Table 4:** Comparison of phase-shift angles φ_0.1_ (°) at 36 test points on the large steel sheet with the blue coating at a measurement frequency of 0.1 Hz.

Coating	1 Layer			2 Layers			3 Layers		
Row/column	1	2	3	4	5	6	7	8	9
1	19.8	26.8	10.1	9.9	16.7	14.2	41.5	40.6	40.9
2	26.3	30.5	11.3	27.7	36.0	32.6	40.4	42.8	30.8
3	38.3	9.3	10.9	39.6	13.0	30.0	41.7	45.0	22.0
4	10.2	54.2	7.4	4.5	33.0	29.1	30.4	82.5	20.0

**Table 5:** Comparison of calculated logarithms of the impedance moduli Z_0.1_ (Ωcm^2^) at 36 test points on the basis of two correlation relationships shown in Figure 15; the percentage difference between the measured and calculated logarithm of the impedance modulus is given in brackets.

Coating	1 Layer			2 Layers			3 Layers		
Row/column	1	2	3	4	5	6	7	8	9
1	6.78 (−3%)	6.74 (+7%)	6.86 (−5%)	8.86 (−14%)	8.71 (−19%)	8.94 (−34%)	9.58 (−8%)	9.58 (0%)	9.01 (+6%)
2	6.63 (+15%)	6.78 (+18%)	7.08 (−8%)	8.29 (+2%)	8.79 (−1%)	8.86 (+1%)	9.47 (+1%)	9.20 (+3%)	9.20 (−8%)
3	7.10 (+21%)	7.13 (+10%)	7.05 (+2%)	8.27 (+15%)	8.27 (−11%)	8.38 (−11%)	8.97 (+8%)	8.58 (+7%)	8.88 (+3%)
4	6.94 (+1%)	6.94 (−12%)	7.30 (−24%)	7.83 (−15%)	7.72 (0%)	7.94 (−16%)	8.77 (−2%)	8.58 (+16%)	8.88 (−1%)

## Data Availability

The data presented in this study are available on request from the corresponding author.
